# Comparison of the oropharyngeal leak pressure in head rotation between a first-generation laryngeal mask and the i-gel laryngeal mask during general anesthesia - a randomized controlled trial

**DOI:** 10.1186/s12871-025-03368-5

**Published:** 2025-09-17

**Authors:** Philipp Brandhorst, Valentina Palella, Konstantin Cloeren, Stephan Voegeler, Sascha Treskatsch, Moritz Weigeldt

**Affiliations:** Department of Anaesthesiology and Intensive Care Medicine, Campus Benjamin Franklin, Charité - Universitätsmedizin Berlin, Corporate Member of Freie Universität and Humboldt Universität zu Berlin, Hindenburgdamm 30, Berlin, D-12203 Germany

**Keywords:** Airway head and neck position laryngeal mask airway i-gel OLP

## Abstract

**Background:**

The laryngeal mask airway (LMA) is essential in anesthesia, using an air-inflatable cuff for optimal pharyngeal fit. The i-gel^®^ LMA (i-gel) is unique for its thermoplastic elastomer material making an air-inflatable cuff unnecessary and has shown higher oropharyngeal leak pressure (OLP) than first-generation LMA (cLMA) in neutral head position, however, mixed results for head rotation. This prospective study aimed to compare OLP between the cLMA and i-gel during head rotation.

**Methods:**

Patients undergoing elective surgery securing the airway with either cLMA or i-gel were included. Exclusion criteria included a Body Mass Index > 30 kg/m², cervical spine pathologies or restricted head rotation. OLP was measured after induction at 60° bilateral head rotation for primary outcome. Secondary outcomes were OLP at 0° head rotation and postoperative complications on postoperative day 1.

**Results:**

116 patients were randomized to either the cLMA (*n* = 60) or i-gel (*n* = 56) group. No significant difference was found in the OLP with 60° head rotation to the left (cLMA 20 cmH_2_O [Interquartile range [IQR] 18–22], i-gel 21 cmH_2_O [IQR 18-22.75], *P* = 0.752) and the right (cLMA 20 cmH_2_O [IQR 18–22], i-gel 20 cmH_2_O [IQR 15–21], *P* = 0.324). OLP was also comparable in a neutral head position (*P* = 0.368). No significant differences in postoperative outcome were observed.

**Conclusions:**

This study demonstrated comparable OLP between cLMA and i-gel during head rotation. This indicates equivalent efficacy and seal integrity of both LMAs during head rotation, resulting in sufficient tidal volumes during rotated head positions.

**Trial registration:**

German Clinical Trials Register, retrospectively registered under DRKS00031785 (date of registration 26.04.2023).

**Supplementary Information:**

The online version contains supplementary material available at 10.1186/s12871-025-03368-5.

## Background

Since its first description in 1983 [1] and commercial introduction in Germany in the 1990 s, the laryngeal mask airway (LMA) has become an essential part of anesthesia practice [2]. In addition, its use in difficult airway and emergency medicine was included in the original descriptions [3], which is reflected in the current guidelines [4, 5]. LMAs are less traumatic and require less time to secure the airway. However, a theoretical disadvantage is gastric insufflation and a consequent risk of aspiration due to lack of endotracheal occlusion, especially at high ventilation pressures and reduced oropharyngeal leak pressure (OLP) [6]. Therefore, in the course of further development, a distinction has been made between classic LMA (first-generation laryngeal mask airway; cLMA) and second-generation LMA. Beside fitting optimization, second-generation LMAs also have an esophageal drainage channel through which gastric contents can be aspirated [7, 8]. The i-gel^®^ mask (i-gel) is a second-generation LMA, with the unique feature of a thermoplastic elastomer material that automatically conforms to the surrounding structure when heated to body temperature [7, 9].

OLP is a clinically important parameter as it provides information on the optimal airway seal and anatomical position of the LMA. The higher the OLP, the tighter the LMA fit and the less secretions or blood from the naso- and oropharyngeal areas can leak into the trachea or cause unwanted insufflation of the gastrointestinal tract [7]. In comparison to a cLMA, the i-gel was shown to have a higher OLP in neutral head position [10].

However, moving the head out of the neutral position can affect the OLP. In this context due to the necessity of surgical access routes, it may also be required to rotate the head, for example, during head and neck surgery (ear, nose and throat surgery, neurosurgery, oral and maxillofacial surgery, ophthalmology). Furthermore, due to the positioning of the patient, there is also iatrogenic pulling during procedures on the upper extremities, which can lead to rotation of the head.

However, there is a lack of literature on how OLP changes with head rotation, and studies have produced conflicting results. One study found no difference in OLP with head rotation for the i-gel [11], while another prospective study showed a decrease in OLP with head rotation for second-generation LMAs and the i-gel [12]. Some minor discrepancies may be due to study conditions; others may be related to the generation or type of LMA used. Interestingly, a meta-analysis of 13 randomized studies found no significant difference in OLP when comparing mainly second-generation LMAs during head rotation. Again, these studies were heterogeneous, including pediatric population, and mainly using neuromuscular blocking agents. Therefore, generalizability is still scarce [13].

Notwithstanding the recommendation of second-generation LMAs due to their superior fit, at least in Germany, first-generation devices are still widely employed due to their cost-effectiveness.

The objective of this prospective study was thus to compare the OLP of a cLMA (Ambu^®^ AuraStraight™) with that of the newer i-gel during head rotation under general anesthesia.

## Methods

### Study design

This single-center, prospective, randomized intervention study was conducted at a tertiary hospital. The study was approved by the ethics committee of the Charité - Universitätsmedizin Berlin (Ethical Committee N° EA2/269/22 of May 2023) and at German Clinical Trials Register (DRKS00031785, date of registration 26.04.2023). Included patients gave their written informed consent. The study protocol was carried out in accordance with the Declaration of Helsinki and the CONSORT-Guidelines for randomized controlled trials.

### Patients and setting

The trial was performed from the 11th of April 2023 and the 28th of June 2023. Adult patients (≥ 18 years) ASA physical status I–III who underwent elective surgery under general anesthesia with an LMA according to institutional standard operation procedures were checked for eligibility. Exclusion criteria included obesity (Body Mass Index, BMI > 30 kg/m²), cervical spine pathologies or restricted head rotation, contraindications to LMA use, and inability to provide consent.

Prior to inclusion, the cervical spine of the awake patient was evaluated for motion restriction, pain, and neurological pathology. Administration of anesthesia and performance of measurements were conducted in the anesthesia induction room of a central operating theater area. Induction of anesthesia was accomplished with the administration of intravenous opioids (fentanyl, sufentanil, and remifentanil) and propofol in accordance with the respective institutional standard operation procedures. Maintenance of anesthesia was carried out as total intravenous anesthesia (TIVA) or balanced anesthesia (sevoflurane). Depth of anesthesia was quantified through processed electroencephalography (Sedline^®^, Masimo Corporation, 52 Discovery Irvine, CA 92618, USA), and the study LMA was placed when the patient status index (PSI) was < 50. LMA type (cLMA: Ambu^®^ AuraStraight™ vs. i-gel) was selected in adherence to the study protocol and prior randomization, and the size of the LMA was determined based on the specifications outlined by the manufacturer. Proper placement of the LMA was confirmed when a stable expiratory CO_2_ curve with values between 35 and 45 mmHg was observed and no detectable air was observed at a fresh gas flow (FGF) of 1 L/min. After LMA insertion a resting period of 5 min was allowed to equilibrate and warm the LMA before measurements began. Subsequently, adjustments were made to the ventilation settings and the cuff pressure of the cLMA was verified to be 60 cmH_2_O. The fraction of inspired oxygen (FiO₂) was set to 1.0 at an FGF of 3 L/min. The ventilation mode was set to pressure controlled. Inspiratory pressure (P_insp_, cmH_2_O), positive end-expiratory pressure (PEEP, cmH_2_O), and expiratory tidal volume (TV, ml) were recorded prior to measurement. Parameters were considered to be within the normal range if the P_insp_ was less than 20 cmH_2_O at a PEEP of 3–5 cmH_2_O with a TV of 8 ml/kg body weight. These parameters were employed as a surrogate for adequate ventilation and correct positioning of both LMAs.

### Primary Endpoint

The primary endpoint was OLP in cm H_2_O in head rotation at 60° left and right, without extension or inversion of the head. OLP was measured by closing the expiratory valve (APL valve, adjustable pressure-limiting valve) to 40 cmH_2_O, setting the FGF to 8 L/min and switching to manual ventilation mode without ventilating. This resulted in a steady state of the ventilation pressure (plateau in the ventilator graph). This numerical value was recorded as OLP by the anesthesia machine (Dräger Medical Primus^®^), the minimum detectable pressure step was 1 cmH_2_O. In addition, it was recorded whether an acoustic leakage of the LMA was perceptible at this time with one member of the study team listening for an audible noise over the mouth [14]. OLP was measured first at 0° and then at 60° head rotation, first to the left and then to the right, with no additional flexion or extension of the head.

All measurements were performed in the order mentioned above. After the initial OLP measurement was finished, the head was rotated, and we waited for the ventilation parameters to reach equilibrium. Once this occurred, the next OLP measurement was performed.

### Secondary Endpoint

Secondary endpoints include the OLP in cmH_2_O at 0° head rotation and postoperative complications such as sore throat, hoarseness, swallowing difficulties, soft tissue damage (lip, tongue, oral and pharyngeal mucosa and larynx) and tooth damage on postoperative day 1. Tidal volume, P_insp_, and PEEP were measured before the start of the study measurements. Postoperative complications were assessed during a study visit on the first postoperative day and documented using our case report form.

### Randomization

Patients were assigned to either cLMA or i-gel group in a 1:1 ratio using digital 4-block randomization. A sealed envelope was used for the allocation concealment method, which was opened by the study team after the patient was anaesthetized immediately before laryngeal mask insertion.

### Sample size

We performed an a priori power analysis based on previous research [10, 12]. This showed a pooled standard deviation (SD) of 6.31327 for the difference between a laryngeal mask with cuff and an i-gel. A 3 cmH_2_O or greater difference in OLP was considered clinically relevant [10]. We calculated that a total sample of 114 patients would be needed to detect a significant difference between study groups for primary endpoint OLP (alpha-level of 5%, 1-β probability of 80%) [15]. To account for dropouts, a study design with a case number of 63:63 was planned.

### Statistical methods

Data are reported as numbers of patients (%), median with 25 to 75th percentiles [IQR]. For univariate analyses of statistical significance, nominally scaled data were analyzed using chi-square test and ratio scaled data using Mann-Whitney test. Testing for normal distribution was carried out using the Shapiro-Wilk test. The level of statistical significance was set at *P* < 0.05. In general, the absence of a statistical difference does not imply formal equivalence.

Statistical analyses were performed with IBM SPSS Version 29.0.1.0; Armonk, New York, United States.

## Results

**Fig. 1:**
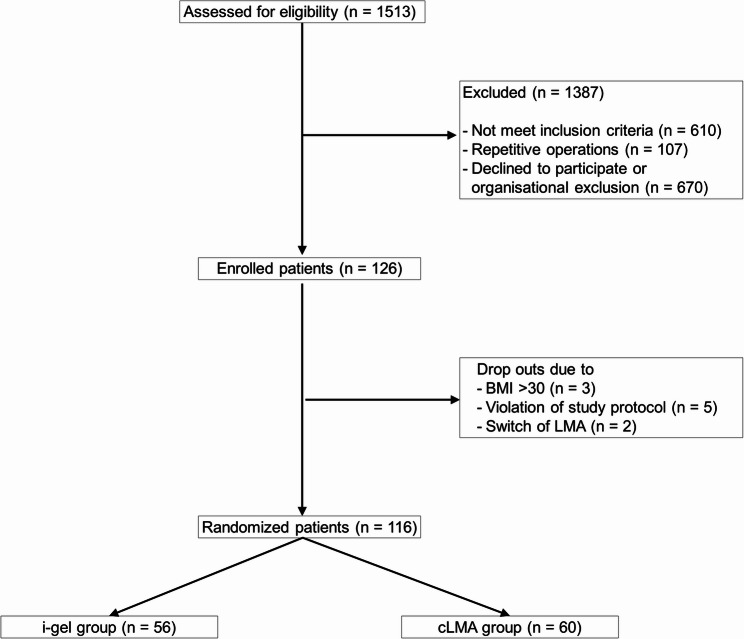
Consort flowchart. *BMI*,* Body mass Index*

A total of 126 patients were enrolled from a cohort of 1513 screened individuals. Of these, 10 patients did not complete the study, and 116 patients were randomly assigned to either the cLMA (*n* = 60) or the i-gel (*n* = 56) study group (Fig. 1). Demographic and baseline parameters did not differ between the study groups and are shown in Table 1.

**Table 1:** Demographic and baseline parameters

	cLMA, *n* = 60	i-gel, *n* = 56
Female, n (%)	20 (33.3%)	17 (30.4%)
Male, n (%)	40 (66.7%)	39 (69.6%)
Age, median [IQR]	60.5 [50–74]	58.5 [40–75]
BMI (kg/m^2^), median [IQR]	24.7 [22–27]	24.1 [23–26]
Urological surgery, n (%)	33 (55%)	26 (46%)
Trauma surgery, n (%)	14 (23%)	12 (21%)
General surgery, n (%)	4 (7%)	8 (14%)
Ophthalmology, n (%)	5 (8%)	4 (7%)
ENT, n (%)	1 (2%)	4 (7%)
Vascular surgery, n (%)	2 (3%)	1 (2%)
Neurosurgery, n (%)	1 (2%)	1 (2%)
TIVA, n (%)	59 (98%)	55 (98%)
Fentanyl, n (%)	34 (57%)	25 (45%)
Sufentanil, n (%)	19 (32%)	24 (43%)
Remifentanil, n (%)	7 (12%)	7 (13%)

*BMI*Body-Mass-Index,* IQR*Interquartile range,* TIVA*Total intravenous anesthesia

**Fig. 2:**
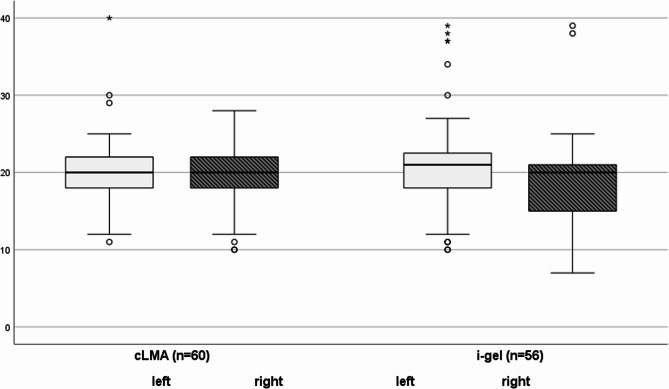
Boxplots showing OLP in cmH2O with 60° rotation of the head for cLMA left, 20 [18-22] cmH2O, right 20 [18-22] cmH2O; i-gel left, 21 [18-22.75] cmH2O and right, 20 [15-21] cmH2O as median [Interquartile range]. Oropharyngeal leak pressure, OLP

There was no significant difference in OLP at 60° head rotation between the first-generation cLMA and the second generation i-gel. (Fig. 2; Table 2).

**Table 2:** Primary and secondary endpoints

	cLMA, *n* = 60 median [IQR]	i-gel, *n* = 56 median [IQR]	*P* value
OLP (cmH_2_O) in neutral head position	21 [19–22]	21 [19–25]	0.368
OLP (cmH_2_O) with left head rotation	20 [18–22]	21 [18-22.75]	0.752
OLP (cmH_2_O) with right head rotation	20 [18–22]	20 [15–21]	0.324
Tidal volume (ml) in neutral head position	491 [420–549]	518 [443–609]	0.181
Tidal volume (ml) with left head rotation	480 [412–557]	485 [414–581]	0.641
Tidal volume (ml) with right head rotation	449 [391–520]	488 [395–566]	0.358
P_insp_ (cmH_2_O) in neutral head position	13 [11–14]	13 [12–15]	0.434
P_insp_ (cmH_2_O) with left head rotation	13 [11–15]	13 [11–15]	0.978
P_insp_ (cmH_2_O) with right head rotation	13 [12–14]	13 [11–15]	0.889
PEEP (cmH_2_O) in neutral head position	5 [3–5]	5 [3–5]	0.399
PEEP (cmH_2_O) with left head rotation	5 [3–5]	5 [3–5]	0.481
PEEP (cmH_2_O) with right head rotation	5 [3–5]	5 [3–5]	0.549

*IQR*Interquartile range,* OLP*Oropharyngeal leak pressure,* PEEP*Positive end-expiratory pressure,* P*_*insp*_ Inspiratory pressure

There was also no significant difference in the OLP in a neutral head position between the two study groups (Table 2).

Concerning P_insp_, PEEP or tidal volume, both groups were comparable. Additionally, patients reported comparable incidences of sore throat, hoarseness, swallowing difficulties, and injuries to the tongue and lips between groups. There were no observed injuries to the oral cavity, larynx, or teeth (Table 3).

**Table 3:** Postoperative complications regarding airway management

Location	cLMA, *n* = 60	i-gel, *n* = 56	*P* value
Total, n (%)	18 (30%)	16 (28.6%)	0.916
Sore throat	6 (10%)	3 (5.4%)	0.352
Hoarseness, n (%)	9 (15%)	6 (10.7%)	0.494
Swallowing difficulties, n (%)	6 (10%)	4 (7.1%)	0.385
Lip, n (%)	4 (6.7%)	3 (5.4%)	0.768
Tongue, n (%)	0	2 (3.6%)	0.141
Mouth, n (%)	0	0	
Larynx, n (%)	0	0	
Teeth, n (%)	0	0	

## Discussion

The aim of this study was to investigate the effect of head rotation during general anesthesia on OLP between the cLMA and i-gel. Our results revealed no statistically significant differences in OLP between the two LMAs. This finding suggests that OLP is independent of LMA type and head rotation. However, it is said that OLP is higher with second-generation LMAs [21].

Consistent with these results, a meta-analysis conducted by Kim and colleagues also showed no statistically significant difference in OLP with head rotation among various other supraglottic airway devices. However, these results may not completely be comparable to our study due to differences in the study conditions, such as the use of neuromuscular blocking agents and the inclusion of a pediatric population [13]. This meta-analysis also included studies that primarily focused on second-generation LMAs. Nevertheless, a decrease in OLP was observed with head extension and an increase in OLP with head flexion.

One study found that OLP was significantly higher using cLMA in the rotated neck position with a mean difference of 2.17 cmH_2_O. Other studies also showed higher OLP during head rotation for one device, but no difference in OLP during head rotation for the other devices. These studies used mainly a second generation LMA and the i-gel [16, 22].

In contrast, the OLP for the i-gel is highly variable; in a 2015 meta-analysis comparing a second-generation LMA with the i-gel, the OLP for the i-gel ranged from 22 (SD ± 3.23) to 35.63 (SD ± 4.84) cmH_2_O [17]. Two other studies showed an OLP of 20.07 (SD ± 2.94) cmH_2_O in non-paralyzed and 27.1 (SD ± 6.4) cm H_2_O in paralyzed patients [10, 18]. These differences in OLP are most likely multifactorial and may depend on the different design and material of the respective LMAs as well as anatomical and patient-related factors.

Russo et al. utilized MRI to assess the anatomical positioning of two second-generation LMAs, including the i-gel. They identifying differences in their interaction with surrounding structures. While their findings suggest potential variations in seal effectiveness, our study did not find evidence supporting inferior seal integrity of the i-gel during head rotation [19].

One noteworthy finding from our study was the comparability in ventilation parameters between the two LMAs despite head rotation. Both LMAs achieved adequate TVs with low P_insp_ even in rotated head positions. This indicates that both LMAs maintain their seal and provide adequate ventilation under challenging conditions such as head rotation during anesthesia.

The lack of significant differences in overall low postoperative complication rates between the two LMAs is consistent with previous studies comparing different supraglottic airway devices. Our findings align with those of Mishra et al. who found no significant disparity in complications between the i-gel and a second-generation LMA [20]. This suggests that while LMAs may vary in design and generation, they offer comparable safety profiles in clinical practice. At least there is a difference in terms of sore throats, hoarseness and difficulty swallowing. However, this was not statistically significant. The head was only rotated for a few minutes. This is also a primary limitation, as a longer duration, which is typical of surgeries requiring head rotation, might produce different results with regard to complications or ventilation quality. It is noteworthy that certain position verification tests (e.g., the ‘bubble test’ or suprasternal notch test), which are applicable to second-generation LMAs including a gastric channel, cannot be used with first-generation LMAs; however, this did not translate into any difference in clinical performance or safety between the devices in our study.

## Limitations

Our study had several limitations. The present project is a single-center study; other sites with different clinical practices may produce different results. Double blinding is also not possible due to the visible nature of the LMA placement and head position. This may have introduced observer bias. Although we believe this did not significantly affect our overall results, future studies with larger sample sizes and rigorous blinding procedures are warranted to further validate our findings. In addition, there was a higher dropout rate in the i-gel group. Systematic error was excluded by the 4-block randomization, so it is possible that protocol violations or lack of experience with the product may have led to a switch to another airway device.

We considered a difference of 3 cmH_2_O to be clinically significant [23]. We wanted to compare the study groups using a common and straightforward anesthesia practice. Therefore, we did not perform fiberoptic assessment, which may have influenced the results. In addition, our measurements always had the same sequence of neutral, left and right side of the head. This could theoretically lead to a carry-over effect. Neuromuscular blocking agents appear to have an impact on OLP. The overall likelihood of higher OLP was increased in patients with paralysis and the effect was more pronounced for the second-generation LMA [12, 24]. However, we did not use neuromuscular blocking agents in this study, which could have an effect on OLP.

Also, the complications observed in our study are within the range of prevalence reported in the literature. We did not adjust the cuff pressure after the head was rotated. Rotation could lead to higher pressure and a higher risk of complications. At least there is a difference in terms of sore throats, hoarseness and difficulty swallowing. However, this was not statistically significant. The head was only rotated for a few minutes possibly presenting a limitation, as a longer duration, which is typical of surgeries requiring head rotation, might produce different results with regard to complications or ventilation quality.

To validate this result, a non-inferiority study design would be necessary, requiring a larger sample size.

## Conclusion

This study demonstrated comparable OLP between the cLMA and i-gel during head rotation. This indicates comparable efficacy and seal integrity of both LMAs during head rotation, resulting in sufficient tidal volumes during rotated head positions. This is especially significant given the cLMA’s superior cost-effectiveness.

## Supplementary Information

Supplementary Material 1.

## Data Availability

The datasets used and/or analyzed during the current study are available from the corresponding author upon reasonable request.

## Abbreviations

LMA: Laryngeal mask airway
cLMA: First-generation laryngeal mask airway (classic)
OLP: Oropharyngeal leak pressure
i-gel: I-gel® mask
FGF: Fresh gas flow
FiO₂: Fraction of inspired oxygen
P_insp_: Inspiratory pressure
PEEP: Positive end-expiratory pressure
TV: Expiratory tidal volume
APL: Adjustable pressure-limiting
SD: Standard deviation
IQR: Inter quartile range
BMI: Body mass index
TIVA: Total intravenous anesthesia
